# Identification of regions in the HOX cluster that can confer repression in a Polycomb-dependent manner

**DOI:** 10.1186/1756-8935-6-S1-P86

**Published:** 2013-03-18

**Authors:** Caroline J Woo, Peter V Kharchenko, Laurence Daheron, Peter J Park, Robert E Kingston

**Affiliations:** 1 Department of Molecular Biology, Massachusetts General Hospital, Boston, MA 02114, USA; 2 Informatics Program, Children’s Hospital, Boston, MA 02115, USA; 3 Harvard Stem Cell Institute, Cambridge, MA 02138, USA; 4 Center for Biomedical Informatics, Harvard Medical School, Boston, MA 02115, USA

## 

Polycomb Group (PcG)-mediated repression is critical for cell fate determination and maintenance of gene expression during embryonic development. However, the mechanisms underlying PcG recruitment in mammals remain unclear since few regulatory sites have been identified. We have identified and characterized two new potential PcG-dependent regulatory elements within the human HOXB and HOXC clusters. Their repressive activities are similar to a previously identified element in the HOXD cluster. The PcG proteins BMI1 and SUZ12 are recruited to a reporter construct in mesenchymal stem cells and confer repression that was dependent upon PcG expression. In addition, JARID2 was observed to localize to these three elements. Interestingly, the requirement for JARID2 is variable at the different regions despite its localization. We conclude that distinct regions of the mammalian HOX clusters can recruit components of the PcG complexes and confer repression and that JARID2 plays a role in the recruitment of PRC2.

